# Re-evaluating statin activity in cancer

**DOI:** 10.18632/aging.101504

**Published:** 2018-07-22

**Authors:** Laura Camacho, Amaia Zabala-Letona, Arkaitz Carracedo

**Affiliations:** 1 CIC bioGUNE, Bizkaia Technology Park, Derio, Spain; 2 Biochemistry and Molecular Biology Department, University of the Basque Country, Bilbao, Spain; 3 CIBERONC, Madrid, Spain; 4 IKERBASQUE, Basque Foundation for Science, Bilbao, Spain

**Keywords:** prostate cancer, statins, cholesterol

Statins are 3‑hydroxy‑3‑methylglutaryl‑coenzyme A reductase (HMG‑CoA reductase) inhibitors, a family of pharmacologic agents widely prescribed for hypercholesterolemia and cardiovascular disease. Cumulative research has aimed at elucidating the impact of statins in cancer risk and progression, with varying results. These agents have been shown to affect several cell and tissue activities, including migration and invasion, proliferation, angiogenesis, apoptosis or cancer stem cell function. Paradoxically, the reported activity of statins ranges from antitumoral effects [1] to the promotion of proliferation and metastasis [2].

Observational studies in prostate cancer (PCa) have yielded inconclusive results, with only a fraction of them reporting a protective effect, whereas others showing no or positive associations [3]. There are various aspects that could act as confounding factors in these studies, including the type of statin, the dose, its capacity to normalize serum cholesterol or co-morbidities and co-existing treatments. In our study, we undertook an experimental approach, in which we aimed at understanding the contribution of statins treatment in the context of obesity, taking advantage of a genetic mouse model of prostate cancer that develops pre-neoplastic lesions [4]. To our surprise, simvastatin administration at doses compatible with human patient anticholesterolemic treatment resulted in the development of prostate cancer in obese mice harboring heterozygous deletion of *Pten*. The increase of prostate cancer aggressiveness upon statin treatment was observed with different compounds, and both *in vitro* and *in vivo*. The fact that the results *in vivo* were more pronounced than in isolated cells suggests that beyond the cell autonomous effects of statins on tumour cells, these compounds could also have an impact on the tumor microenvironment.

The research around the activity of statins in cancer has been influenced by the doses of statins employed. Micromolar doses of statins consistently suppress tumor cell survival and proliferation [4]. However, a physiologically relevant dose of statins has been less explored in cancer research, thus justifying the aforementioned discrepancies [4–6]. We found that, as opposed to micromolar concentrations, nanomolar doses of statins were not cytotoxic in various prostate cancer cell lines, and rather increased anchorage-independent growth *in vitro*.

Clinical studies have predominantly focused their attention on the association of statins to the risk of developing cancer (or specific tumor incidence). However, less is known about the impact of these compounds on the aggressiveness of pre-existing tumors. Consistent with previous data, we found out that treatment with statins was associated with a significant reduction in PCa risk. Interestingly, in this same cohort, patients with PCa taking statins presented a higher incidence of high aggressiveness tumors.

As mentioned earlier, the most robust effect of statin administration was seen in obese mice. In line with our results in mice and patients, a population-based case–control study (where statin use was not associated with overall PCa risk) reported an increased risk associated with statin use among obese men (BMI ≥30 kg/m2), with a stronger association in long-term treated individuals [7]. These results provide a new biological perspective in the field of statins, with important implications for human health. The molecular grounds explaining the conflicting activity remain obscure. Therefore, the experimental models that recapitulate many of the undesired effects of statins should be further exploited for molecular cancer research. In this context, the in vivo dimension and a competent immune system could essential aspects to provide a full image for this biological conundrum.
